# Towards efficient motor imagery interventions after lower-limb amputation

**DOI:** 10.1186/s12984-024-01348-3

**Published:** 2024-04-15

**Authors:** Elodie Saruco, Arnaud Saimpont, Franck Di Rienzo, Benjamin De Witte, Isabelle Laroyenne, Fanny Matéo, Marion Lapenderie, Sarah Goutte Solard, Isabelle Perretant, Charlotte Frenot, Philip L. Jackson, Aymeric Guillot

**Affiliations:** 1 Universite Lyon 1, LIBM, Laboratoire Interuniversitaire de Biologie de la Motricité, UR 7424, Villeurbanne, F-69622 France; 2 Centre Médico-Chirurgical de Réadaptation des Massues - Croix-Rouge française, 92 rue Dr. Edmond Locard, Lyon Cedex 05, 69322 France; 3 École de Psychologie, Université Laval, Centre Interdisciplinaire de Recherche en Réadaptation et Intégration Sociale (CIRRIS), Quebec, Canada

**Keywords:** Mental practice, Amputees, Clinical rehabilitation, Motor recovery

## Abstract

**Background:**

The therapeutic benefits of motor imagery (MI) are now well-established in different populations of persons suffering from central nervous system impairments. However, research on similar efficacy of MI interventions after amputation remains scarce, and experimental studies were primarily designed to explore the effects of MI after upper-limb amputations.

**Objectives:**

The present comparative study therefore aimed to assess the effects of MI on locomotion recovery following unilateral lower-limb amputation.

**Methods:**

Nineteen participants were assigned either to a MI group (*n* = 9) or a control group (*n* = 10). In addition to the course of physical therapy, they respectively performed 10 min per day of locomotor MI training or neutral cognitive exercises, five days per week. Participants’ locomotion functions were assessed through two functional tasks: 10 m walking and the Timed Up and Go Test. Force of the amputated limb and functional level score reflecting the required assistance for walking were also measured. Evaluations were scheduled at the arrival at the rehabilitation center (right after amputation), after prosthesis fitting (three weeks later), and at the end of the rehabilitation program. A retention test was also programed after 6 weeks.

**Results:**

While there was no additional effect of MI on pain management, data revealed an early positive impact of MI for the 10 m walking task during the pre-prosthetic phase, and greater performance during the Timed Up and Go Test during the prosthetic phase. Also, a lower proportion of participants still needed a walking aid after MI training. Finally, the force of the amputated limb was greater at the end of rehabilitation for the MI group.

**Conclusion:**

Taken together, these data support the integration of MI within the course of physical therapy in persons suffering from lower-limb amputations.

**Supplementary Information:**

The online version contains supplementary material available at 10.1186/s12984-024-01348-3.

## Introduction

There is now compelling evidence that both actually performing and mentally simulating a movement (motor imagery [MI]) are mediated by overlapping neural networks including motor regions [1]. Such neurofunctional equivalence between actual execution and corresponding MI has been observed for simple manual tasks [2, 3] and complex motor sequences involving the lower limbs [4]. Several studies provided evidence that most of the active brain regions involved in the physical performance of locomotor tasks were also recruited during MI (for a review, see [4]). Interestingly, the neural plasticity resulting from motor learning [5] has been observed as a result of MI interventions [6–8], hence highlighting the relevance of incorporating MI interventions in the classical course of physical therapy and motor (re)learning paradigms (for reviews, see [9–11]). The field of neuroscience has witnessed a huge interest in the exploration of MI as a relevant tool for neurocognitive stimulation in patients suffering from brain disease. By engaging mental simulations of actions, MI is further likely to activate brain regions associated with attention, memory, and decision-making. This multifaceted engagement suggests that MI goes beyond the motor domain, influencing broader cognitive functions and contributing to a neurocognitive synergy. The cognitive stimulation provided by MI might therefore not only complement physical rehabilitation but also contribute to the restoration of motor function by harnessing inherent brain plasticity.

The independent ability to move is a determinant of satisfying quality of life [12]. Thus, locomotor recovery is a critical issue after lower limb motor damage [13]. People who have lower limb amputation (LLA) often present severe impairment of locomotor capacities requiring extensive rehabilitation [14, 15]. When physical practice is not possible on the grounds of high fatigability or impossibility to move, MI represents a cost-effective adjunct rehabilitation strategy [16]. Despite the positive effects observed on the recovery of locomotor capacities in persons suffering from central nervous system impairments, such as stroke or Parkinson Disease [17–19], research on the efficacy of MI interventions after peripheral nervous system injury such as LLA remain scarce. Interestingly, there is converging evidence supporting that the ability to perform accurate MI after amputation is preserved. In a sample of upper-limb amputees, Raffin et al. [20] showed that the overlapping of the networks underlying MI and corresponding actual execution was preserved following upper limb amputation, and that this pattern was distinct from the one recruited during attempts to move the phantom limb. The same authors further validated both the relative preservation of MI vividness and its temporal congruence with actual movement times (ability to match actual execution time during MI) [21]. Spurred by these findings, pioneering experimental studies explored the possibility for persons with LLA to perform MI [22, 23]. These studies showed that, although MI vividness and temporal accuracy were affected, particularly for impaired movements, MI scores were preserved and rapidly increased with the use of the prosthesis for both single-joint [23] and locomotor movements [22]. Considering the influence of MI accuracy and vividness on subsequent performance improvement, the overall preservation of MI ability following amputation certainly lies at the foundation of promising therapeutic effects of MI in persons with LLA.

Further evidence to support the use of MI in amputees comes from a functional magnetic resonance study designed to explore the organization of the somatosensorial cortex of persons with upper limb amputation that revealed the beneficial effect of a six-week MI program [24]. The authors reported that the cerebral changes elicited by both relaxation (body-scan) and MI of the phantom limb counteracted the maladaptive remapping of the sensorimotor cortex [25] and led to a significant decrease of phantom-limb pain. Beneficial effects of MI in persons with LLA were also found. Cunha et al. [26] observed a significant improvement of the ground reaction force following a four-week locomotion-oriented program, each week including actual gait training combined with three MI sessions of 40 min, during which participants imagined sitting down and raising from a chair, walking through different modalities (e.g., fast, up and down a staircase, up and down a ramp), running or jumping over obstacles. However, despite the large incidence of transfemoral amputations [27], only persons with a transtibial amputation and whom surgical procedure was performed one to four years before enrollment, were included in this study. One study specifically investigated the therapeutic potential of MI in persons with transfemoral amputation, albeit in the context of a single-case design, and seven years after surgery [28]. Spanned over four weeks, the functional training program of the lower limbs included MI of walking, balancing, and reaching exercises, in the absence of any physical practice. The authors reported a significant decrease of phantom-limb pain, and the possibility to walk a short distance alone, following a 12-session MI program.

Overall, biomechanical data supported some functional improvement after MI training [26], and preliminary clinical findings reinforced this assumption and the potential benefits of MI after LLA [28]. However, such effects of MI have only been studied in participants at a chronic stage, several years after amputation, hence leaving open the question as to the potential effects of mental training directly after surgery. The present study was therefore specifically designed to explore, for the first time, the therapeutic benefits of implementing MI during the entire course of physical therapy, i.e., from the acute phase to an independent degree of mobility, in persons suffering from transtibial and transfemoral LLA. We expected that MI training would promote recovery of locomotor-related skills by enhancing functional capacities like walking speed and dynamic balance. The effect of MI on the amputated limb force and on the level of pain were also investigated.

## Methods

### Participants

**Fig. 1:**
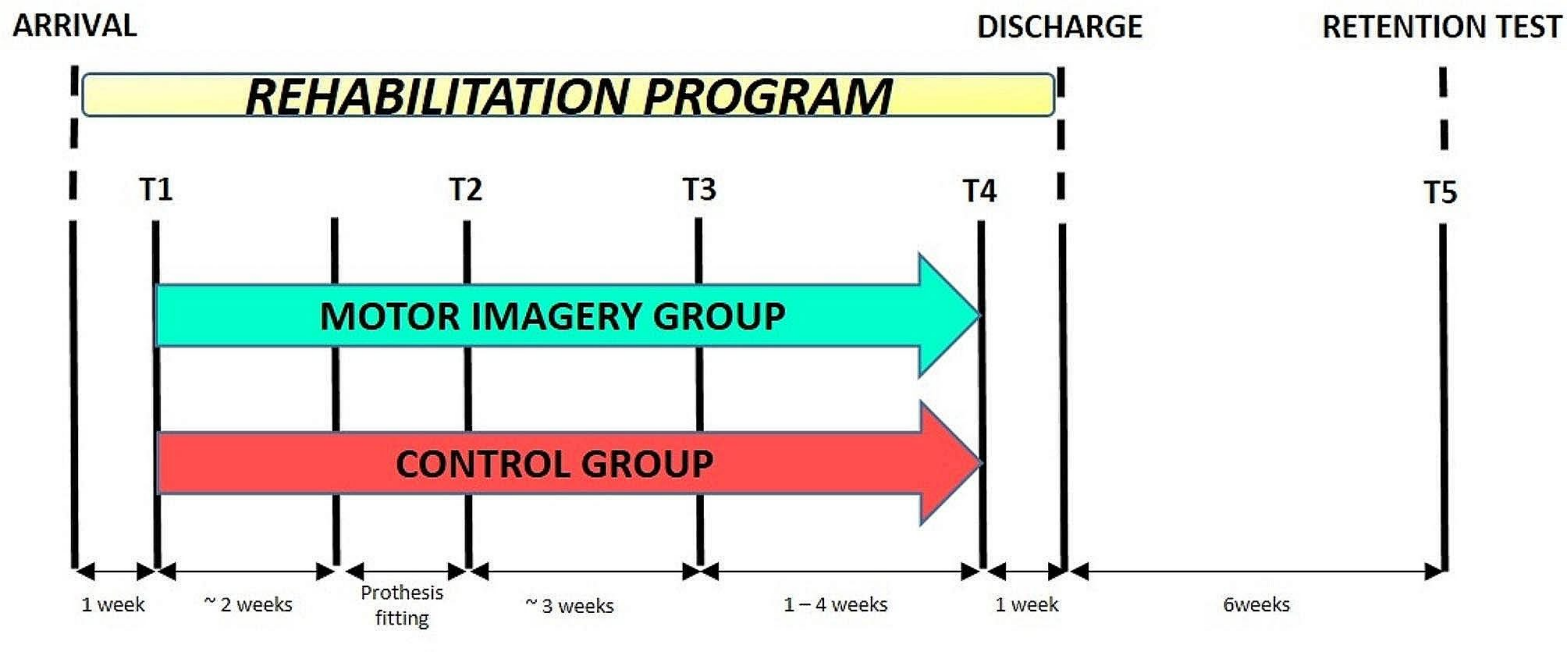
Experimental design showing the chronology of assessments. One week after arrival at the rehabilitation center (T1), participants were assigned to the MI or control group and started to perform MI or the control task concomitantly with physical therapy, up to one week preceding the end of the rehabilitation program (T4). Participants were tested at their arrival to the center (T1), after prosthesis fitting once they could walk with it safely (T2), at an equivalent time of rehabilitation (T3), at an equivalent functional rehabilitation level before leaving the center (T4), and six weeks after the end of the rehabilitation program (T5)

Twenty-two persons with LLA, recruited in the “Centre Médico-Chirurgical de Réadaptation des Massues - Croix-Rouge Française” (Lyon, France), voluntarily participated in the present study that was approved by the local ethics committee (2015-A00573-46). Due to personal relocation, one participant did not complete the study after moving to another region, and two participants quit voluntarily after losing interest in the experiment. The characteristics of the 19 participants who fully completed the protocol and were included in the statistical analysis are reported in Table 1. Inclusion criteria were the following: *(i)* being included in the rehabilitation program of the “Centre Médico-Chirurgical de Réadaptation des Massues” *(ii)* being aged between 18 and 80 years old, *(iii)* having, during the last 20 days, suffered from an unilateral transfemoral or transtibial amputation of vascular, trauma, infectious or cancerous origin, *(iv)* having a score above 24/30 on the Mini-Mental State Examination [29], and *(v)* currently not participating to any other research protocol. Exclusion criteria included the presence of *(i)* neurologic and/or psychiatric disorders, *(ii)* motor dysfunctions unrelated to the present amputation, and *(iii)* guardianship, as well as any other administrative or legal right deprivation measure. After the validation of participation by the physician, participants signed an informed consent form. All participants arrived at the rehabilitation center maximum two weeks after their amputation and were included in the study within the week upon their arrival at the rehabilitation center (Fig. 1). For medical facilitation, participants were continuously assigned in a predetermined group depending the order of inclusion (the Control group was first completed, before the MI group, to avoid any contamination across the participants). The MI and control groups entailed nine and ten participants, respectively, and the analysis strategy was “intention to treat”. The Trend statement complementing the widely adopted consolidated standards of reporting trials was used in this study [30].

**Table 1:** Participants’ characteristics

Participant	Age	Sex	Dom. Side	Ethiology	Level	Prosthetic knee	Prosthetic foot
CTRL#1	55	M	YES	Cancerous	TT		K-Level II
CTRL#2	66	M	YES	Infectious	TF	3r60	K-Level II
CTRL#3	51	M	NO	Infectious	TT		K-Level II
CTRL#4	57	F	YES	Cancerous	TT		K-Level I
CTRL#5	73	M	NO	Vascular	TT		Articulated
CTRL#6	60	M	NO	Infectious	TT		Articulated
CTRL#7	69	F	YES	Vascular	KD	Locking knee	Articulated
CTRL#8	61	M	YES	Infectious	TF	Hydraulic	K-Level II
CTRL#9	63	M	YES	Infectious	TT		Articulated
CTRL#10	55	F	NO	Vascular	TF	Rheo	K-Level I
MI#1	70	M	YES	Vascular	TT		K-Level II
MI#2	47	M	YES	Traumatic	TT		K-Level III
MI#3	44	F	YES	Infectious	TF	3r60	K-Level III
MI#4	50	M	NO	Cancerous	TF	C-Leg4	K-Level I
MI#5	39	M	YES	Vascular	TT		K-Level I
MI#6	47	M	YES	Traumatic	TF	C-Leg4	K-Level III
MI#7	54	M	NO	Infectious	TT		K-Level II
MI#8	54	M	YES	Traumatic	TF	3r60	K-Level III
MI#9	49	M	YES	Traumatic	TT		K-Level II

CTRL = control. Dom. Side = dominant side amputated. F = female. KD = knee disarticulation. M = male. MI = motor imagery. TF = transfemoral. TT = transtibial.

### Experimental design

#### Rehabilitation program

After their arrival at the rehabilitation center, participants started the rehabilitation program under the supervision of physio- and occupational therapists, five times per week, until they reached independent steady gait and functional balance. As a first step, they received stump care and followed the pre-prosthetic rehabilitation program, mainly composed of static balance exercises. During this early phase, only a small amount of the rehabilitation work was organized around dynamic exercises, mainly consisting in unipodal walking between parallel bars. The same program was scheduled in the two groups, with a daily time of training of about 30 min. After healing was considered satisfactory by the medical team, participants were fitted with a prosthesis. Prosthetic rehabilitation program consisted in 10 min of static exercises and a series of dynamic balance and walking-related exercises per day, depending on the fatigue and pain level of the participant. While the time window usually recommended by the medical team during this period ranges from 30 min to 2 h, we restricted the variability in the duration of these exercises to avoid significant diversity among patients (30 to 45 min).

### Motor imagery practice

Throughout the course of physical therapy, participants from the MI group followed a MI intervention. During break times of locomotor training, they were asked to mentally rehearse the movements they just physically performed, using both visual and kinesthetic modalities of MI, from a first-person perspective. Participants’ ability to perform visual and kinesthetic MI was measured after each trial using a 5-point Likert scale graduated from 1 (no image/no sensation) to 5 (image as clear as seeing/sensation as intense as when physically performing the action). To promote MI accuracy, MI trials were scheduled right after physical practice [31, 32]. Each MI trial consisted in repeating for 2 min the movements previously performed. Practically, during the pre-prosthetic phase, MI training included the mental rehearsal of hip flexion/extension movements alternated with unilateral walking between the parallel bars, with an emphasis on strong and safe ground contact with the intact limb. While it is possible to imagine a motor sequence that is not yet physically feasible, based on the memory of these movements, the decision not to perform such imagery was related to the fact that amputees do not recover their normal locomotor function. Functional recovery and restoration of locomotion abilities will depend on the integration of a prosthesis. It was therefore critical for gait imagery to incorporate the use of this individualized prosthetic device before considering its fine-tuned use. During the prosthetic phase, additional MI trials of the Timed Up and Go test (TUG) [33] were also performed. Participants completed 5 trials per day, so a total of 10 min of MI, 5 days per week. Participants from the control group performed a neutral cognitive task (crossword puzzles and Sudoku games) during an equivalent amount of time, and in the presence of the same physiotherapist.

### Measures

A total of five tests were scheduled (Fig. 1). A first test (T1) was programmed one week after participants’ arrival to the rehabilitation center. A second test (T2) was scheduled after they were fitted with the prosthesis and able to walk continuously during 10 min (including short rest breaks). Due to recovery time variability between participants, a third evaluation (T3) was planned 3 weeks later, to provide “time equivalent” data. As the standardization of the therapeutic patient management was not possible, the program evolution remained somewhat patient-dependent, most especially regarding decisions related to the temporal frame. Consequently, to avoid significant disparities among patients, we established a comparison at T3 based on a similar treatment duration, allowing a comparison for an equivalent duration of rehabilitation. Then, to extend data analysis over a longer period, we conducted an assessment at equivalent functional levels prior to clinic discharge (T4). This choice of two complementary evaluations ensured a more detailed and comprehensive comparison of recovery effects in patients, allowing for an assessment of functional recovery dynamics, i.e. satisfying levels of gait speed and balance (standing up, standing, and sitting without external assistance). Finally, a retention test (T5) was scheduled 6 weeks post-departure from the rehabilitation center. The medical assessors who determined the end-points, as well as the therapists performing the evaluation measures, were blinded and did not have information about whether participants were assigned in the control or MI group.

### Motor imagery ability

To assess participant’s ability to imagine movements, MI vividness and temporal accuracy were measured at T1. MI vividness was assessed through an adapted version of the Kinesthetic and Visual Imagery Questionnaire (KVIQ [34]) focusing on five movements involving lower limbs (see [22] for greater details). For each imagined movement, participants physically performed the sequence beforehand. They scored from 1 (no image/no sensation) to 5 (image as clear as seeing/sensation as intense as when physically performing the action) the clarity/intensity of the images/sensations perceived during MI. The temporal accuracy of MI was assessed with the chronometric measure of a single-joint movement [35]. The time required to actually and mentally perform five consecutive hip abductions was recorded for both intact and amputated sides, with an electronic digital stopwatch (Extech Instruments, model 365,515, USA). The ratio between mean MI and actual execution times was then calculated. The closer the ratio to 1, the better the temporal accuracy of MI.

A manipulation check was scheduled each week to control adherence of the participants to the imagery guidelines, and the quality of their imagery experience. We gathered informal information participants was likely to share about his/her imagery experience, and further asked them to rate the quality of their imagery and describe any difficulty they may have experienced. Very few patients sporadically reported some trouble during the imagery exercises, hence supporting that they overall quite easily followed the instructions as intended.

### Motor recovery

After prosthesis fitting, motor recovery was assessed with two locomotor functional tasks (at T2, T3, T4 and T5): *(i)* the 10 m walking task [36] which required to walk a 10 m distance at a safe speed, and *(ii)* the TUG task where participants get up from a chair, walk 3 m, turned around, came back to the chair, turned around and sat down. During all evaluations, participants could use one or both of their crutches or even a walker, if required for safety reasons. A functional level score and the estimated force of the amputated limb were further collected. These two tests are part of a standardized clinical procedure in patient’s care, and thus frequently used by therapists. The functional score was rated by the medical assessors at T2 and T4. Such score allows healthcare professionals to categorize the level of assistance required for walking, ranging from complete dependence on a wheelchair to no needed assistance. Practically, a 6-level scale here reflected the required assistance for walking (necessity of using a wheelchair, the parallel bars, a walker, a rollator, two walking canes, one walking cane, or no needed assistance). It then aids in treatment planning, and ensures a consistent understanding of patient’s mobility, promoting effective care and support tailored to individual needs. The force of the amputated limb was also measured at T2 and T4, using a 5-level of pressure scale exerted by the physiotherapist. This scale helped to quantify the force provided by the patients against a constant pressure exerted on the residual limb by the therapist. Beside the measurement of the force per se, this force provided relevant information to the therapists to tailor the prosthetic device to individual needs and reduce discomfort. Finally, the time of hospitalization was collected to compare delays separating the different time measurements.

### Pain

Although this experiment was specifically designed to assess the benefits of MI on functional recovery, the potential influence of the intervention on phantom limb pain was also measured. During each of the five tests, participants were asked to rate on an analog scale, ranging from 0 (no pain at all) to 100 (worst pain possible), the intensity of the phantom limb pain they experienced during the last 24 h.

### Group data analysis

The durations of the locomotor-related tasks (10 m walking and TUG) at the different evaluations (T2 to T5), as well as the individual time of hospitalization in days at each evaluation (T2 to T5), were the primary dependent variables quantifying sensorimotor recovery. As secondary outcomes, we also analyzed the clinical assessments of force on the amputated limb on a 5-point Likert-type scale at T2 and T4, as well as pain scores at T1-T5, as subjective dependent variables. KVIQ score and MI_RATIO_ were the dependent variables indexing MI ability. Since MI ability was not measured in the Control group, who did not perform MI training after receiving their prosthesis, we conducted a univariate analysis of KVIQ scores and MI_RATIO_.

We used R [37] and *nlme* [38] to run a linear mixed effects analysis, with by-subject random intercepts, of the dependent variables quantifying motor performance during the prosthetic phase (TUG and walking test durations). We built a random-regression coefficient ANCOVA model testing for the fixed effects of GROUP (MI, Control) and TEST (T1-T5), with interaction term. To account for baseline differences, we included performance at T2 as the covariate. Due to deviations from normality (visual inspection of Q-Q plots), we ran a non-parametric analysis of hospitalization time data using the *ARTool* [39] package. Aligned-Rank Transformation (ART) consists in a preliminary step of data alignment based on the mean estimates of main/interaction effects of a given factorial model, followed by rank assignment [40]. We applied the ART to both linear and linear mixed effects models (with by-subjects random intercept accounting for repeated measures), using the fixed effects of GROUP (MI, Control) and TEST (T1-T5), with interaction term. We obtained partial coefficients of determination (η^2^_P_) as measures of effect sizes, using the ad hoc procedure for linear mixed effects models implemented from the *effectsizes* package [41]. As post-hoc investigations, we used contrast tests of marginal means estimates implemented from the *multcomp* package [42]. The proportion of participants’ distribution across 6 progressive impairment walking categories at T4, corresponding to the end of hospitalization, was compared between MI and Control groups using a Chi-squared independence test. The statistical significance threshold was set up for a type 1 error rate of 5%. Holm’s sequential corrections for multiple comparisons were applied to control the false discovery rate [43].

## Results

### Baseline measures

Physical performance measures were not possible at the beginning of the experiment, nor at T1, since participants were not yet fitted with their prosthesis and could not perform locomotor tasks. Homogeneity of groups at baseline was controlled by considering individual characteristics including force of the amputated leg, stump length and anthropometric measures (allowing calculation of the body mass index), as well as qualitative variables including origin of trauma, dominant/non-dominant side of the amputation, type of the amputation (transfemoral vs. transtibial), gender, and functional level (six levels of performance reflecting the ability to walk with/without assistance). The non-parametric analysis revealed an absence of statistically significant baseline difference for all quantitative and qualitative variables between the groups (Table 2), except age (*p* = 0.03).

**Table 2:** Baseline quantitative and qualitative measures at T2

	Control group	Motor imagery group		Statistical significance
*Quantitative variables* *(Median values ± IQR)*				
Age (years)	61 ± 6.88	53 ± 8.75		W = 65, *p* = 0.03*
Body mass index (kg/m²)	25.89 ± 2.93	25.24 ± 3.66		W = 24, *p* = 0.73
Force of the amputated leg (score/5)	4.00 ± 1.00	4.00 ± 1.00		W = 46.5, *p* = 0.58
Stump length (transtibial)	18.00 ± 1.00	18.00 ± 3.00		W = 10, *p* = 0.67
Stump length (transfemoral)	35.00 ± 9.75	42.50 ± 4.25		W = 2.50, *p* = 0.28
Functional level *(score/6–6 levels of walking with/without assistance)*	2.56 ± 2.00	3.11 ± 2.00		W = 30, *p* = 0.36
*Qualitative variables (%)*				
Gender *(Males/Females)*	70/30		89/11	χ2 = 0.20, *p* = 0.66
Origin of the amputation *(Cancer/infection/traumatic/vascular)*	20/50/-/30		11/22/45/22	χ2 = 5.78, *p* = 0.12
Side of the amputation *(Dominant/non-dominant)*	60/40		80/20	χ2 = 0.11, *p* = 0.74
Type of amputation *(Transtibial/transfemoral)*	40/60		67/33	χ2 = 0.01, *p* = 0.99

### Motor imagery ability

The mean (SD) total KVIQ score of participants in the MI group was 3.49 (1.43), visual and kinesthetic scores being 4.01 (1.19) and 2.98 (1.46). None of them reported a score < 2, supporting their ability to form accurate images/sensations of movements, albeit sometimes blurred for some participants. The mental chronometry ratio was 1.06 (0.13), which indicates close temporal congruence between actual and imagined actions. Finally, average MI vividness score during MI training was 3.22 (0.97), hence indicating that participants were able to form accurate visual and kinesthetic MI.

### Sensorimotor recovery

#### Pre-prosthetic phase

Results at T2 revealed that the main GROUP effect affected walking durations (η^2^_P_ = 0.24, F_(1,17)_ = 5.63, *p* = 0.02), but not TUG durations (η^2^_P_ < 0.01, F_(1,17)_ = 0.09, *p* = 0.76). Walking performance in the MI group [21.06 s (6.30)] was higher than that in the Control group [31.83 s (12.21); *p* = 0.02], whereas TUG durations were similar in both groups [MI: 30.92 s (11.99), Control: 34.29 s (32.40)].

### Prosthetic phase

#### Locomotor performance

The raw durations for the walking test and TUG are provided in Table 3.

**Table 3:** Raw durations (s) across groups and repeated measures (T2-T5) of the prosthetic phase

Mean (SD) task durations (s)				
*CONTROL GROUP*				
	**T2**	**T3**	**T4**	**T5**
10 m Walking	31.83 (12.21)	23.48 (8.53)	22.17 (10.31)	15.61 (6.52)
TUG	30.92 (1.99)	26.68 (11.47)	29.76 (15.23)	18.85 (7.87)
*MOTOR IMAGERY GROUP*				
	**T2**	**T3**	**T4**	**T5**
10 m Walking	21.07 (6.30)	11.89 (2.60)	10.04 (0.84)	9.10 (2.34)
TUG	23.58 (4.42)	15.46 (5.67)	12.54 (1.34)	11.91 (3.03)

**Fig. 2:**
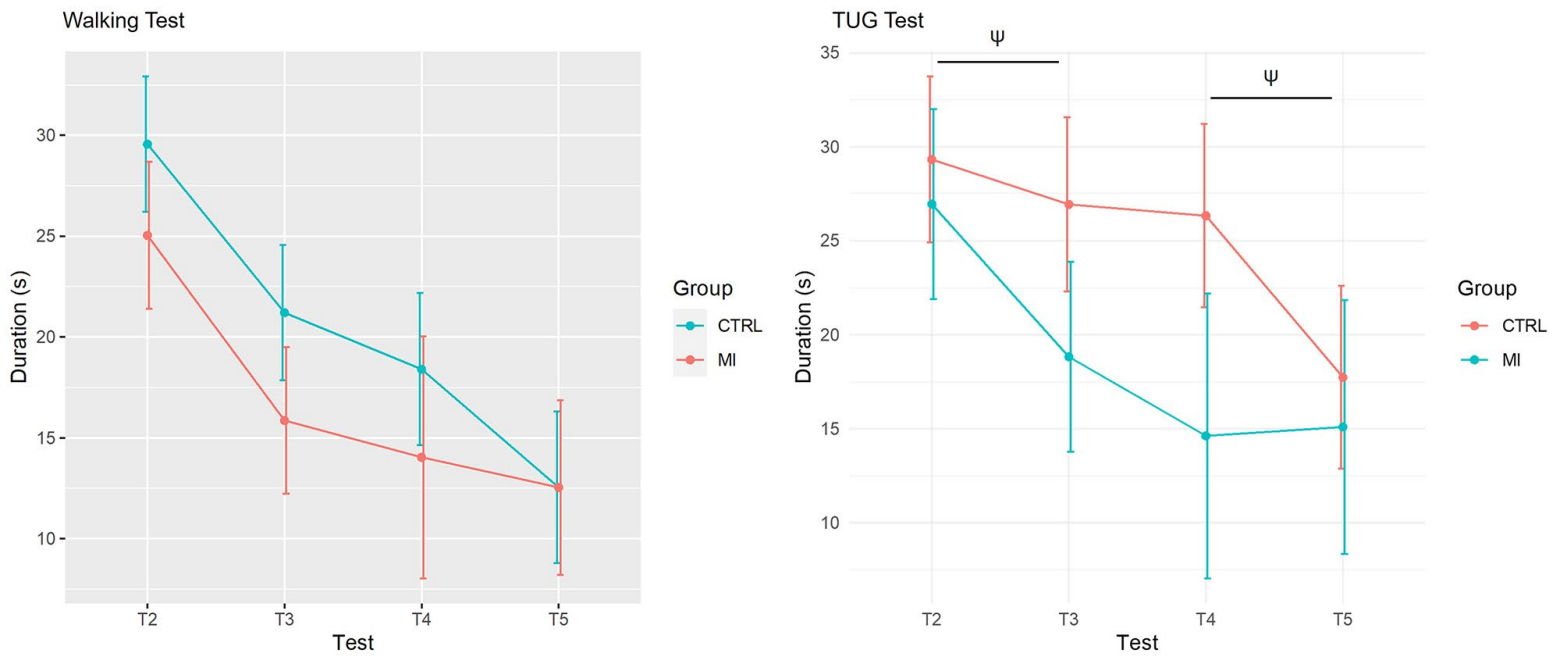
Mean (SD) task duration values (fitted estimates) for the control and MI groups. A- 10 m walking. B- TUG tasks. T = test. TUG = Timed Up and Go. CTRL = Control. MI = Motor Imagery

The ANCOVA revealed that the TEST × GROUP interaction effect for the TUG fell short from the statistical significant threshold for a moderate effect size (η^2^_P_ = 0.13, F_(3,34)_ = 1.78, *p* = 0.08; Fig. 2). There was however no TEST × GROUP interaction effect for the 10 m walking durations (η^2^_P_ = 0.07, F_(3,38)_ = 0.94, *p* = 0.43; Fig. 2). Noteworthy, TUG performance improvements in the MI group between T2 and T3 were marginally greater than those recorded between T2 and T3 in the Control group (*p* = 0.06; Fig. 2). By contrast, performance gains between T4 and T5 in the Control group were marginally higher than the corresponding difference in the MI group (*p* = 0.09, Fig. 2). Both groups exhibited a comparable pattern of improvement on the walking test (Fig. 2).

The ANCOVA also revealed a main TEST effect for the 10 m walking test (η^2^_P_ = 0.68, F_(3,38)_ = 27.16, *p* < 0.001) and TUG (η^2^_P_ = 0.48, F_(3,34)_ = 10.45, *p* < 0.001) tasks. A main GROUP effect was also observed for the 10 m walking test (η^2^_P_ = 0.70, F_(1,16)_ = 37.09, *p* < 0.001) and the TUG (η^2^_P_ = 0.53, F_(1,15)_ = 26.51, *p* = 0.001). Noteworthy, task durations recorded during T2 predicted the durations recorded across the repeated measures of the design, for both the walking [η^2^_P_ = 0.76, + 0.58 s (0.71), *p* < 0.001] and TUG [η^2^_P_ = 0.64, 0.68 s (1.14), *p* < 0.001] tests.

**Fig. 3:**
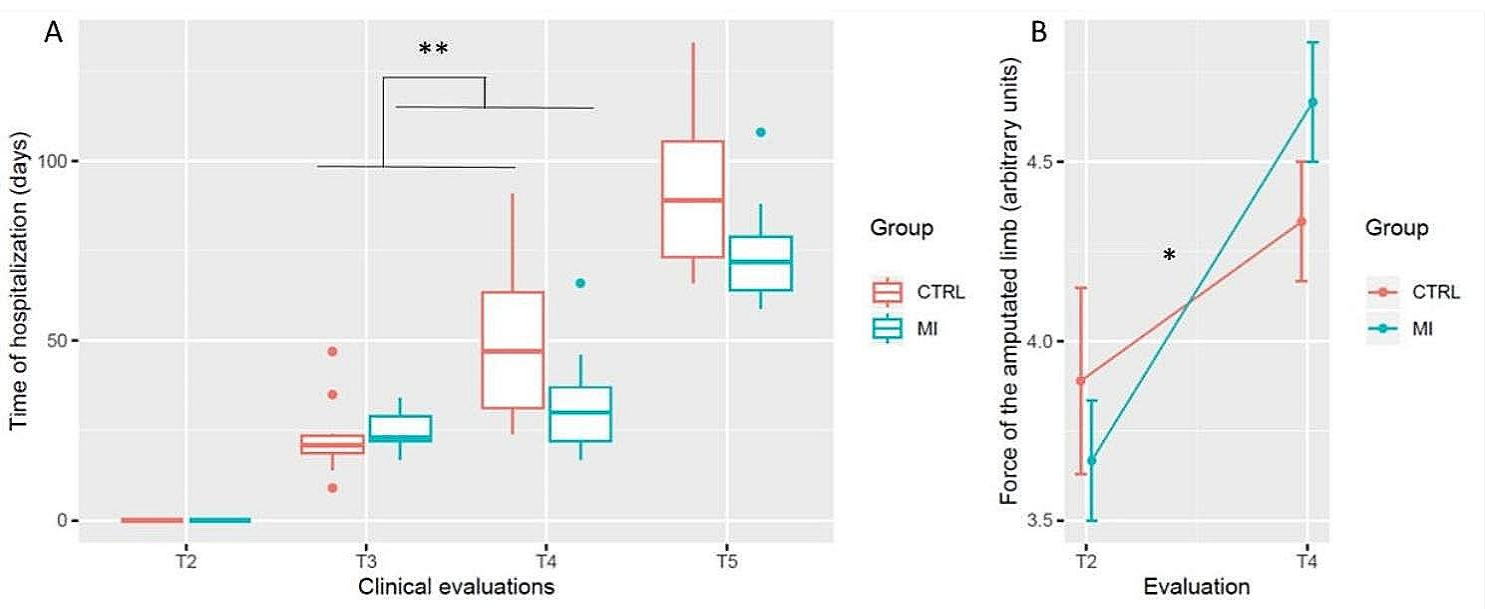
Time of hospitalization (Panel A) and force of the amputated limb (Panel B). CTRL = Control. MI = Motor Imagery

Hospitalization time in days was affected by the TEST × GROUP interaction (η^2^_P_ = 0.24, F_(3, 51)_ = 5.39, *p* = 0.002), as well as by the main effect of TEST (η^2^_P_ = 0.92, F_(3, 51)_ = 199.45, *p* < 0.001). Post-hoc analyzes revealed that while both groups had a similar delay between T2 and T3 (*p* > 0.99), the MI group had a reduced delay separating T3 from T4 (*p* = 0.009, Fig. 3A). The delay between T4 and T5 was similar in the MI and the Control groups (*p* > 0.99). All delays in days differed from each other from T2-T5, irrespective of the group (*p* < 0.001).

Functional level and force of the amputated limb.

The Chi-squared test of homogeneity on the functional level of participants when leaving the hospital was close from the statistical significance threshold (X-squared_(4)_ = 7.2, *p* = 0.06). Control group had reduced proportion of participants walking without any assistance compared to the MI group (0% vs. 16%). The Control group had a greater proportion of participants walking with the assistance of parallel bars and the supervision of a physiotherapist compared to the MI group (33% vs. 11%).

The linear mixed effects analysis with ART revealed a main TEST effect for the force of the amputated limb (η^2^_P_ = 0.67, F(1, 16) = 32.42, *p* < 0.001), while the main GROUP effect was not significant (η^2^_P_ < 0.01, F_(1, 16)_ = 0.08, *p* = 0.77). Data further revealed a TEST × GROUP interaction for the clinical measures of force of the amputated limb (η^2^_P_ = 0.23, F_(1, 16)_ = 4.68, *p* = 0.04; Fig. 3B). Accordingly, improvement from T2 to T4 observed in the MI group (T2: 3.67 (0.5), T4: 4.67 (0.5)) outperformed those recorded in the Control group (T2: 3.88 (0.78), T4: 4.33 (0.5), *p* = 0.04).

#### Pain

Pain ratings were not affected by the main effects of TEST (η^2^_P_ = 0.04, F_(4,56)_ = 0.54, *p* = 0.70), GROUP (η^2^_P_ = 0.05, F_(1,18)_ = 0.85, *p* = 0.37), or the TEST × GROUP interaction (η^2^_P_ = 0.08, F_(4,56)_ = 1.28, *p* = 0.15). Participants’ pain scores in the Control group were 25.00 (26.87) at T1, 23.00 (25.52) at T2, 27.00 (34.25) at T3, 10.63 (17.41) at T4, and 10.00 (14.14) at T5. Respective pain scores for the MI group were 20.56 (31.47) at T1, 13.89 (26.43) at T2, 11.11 (23.15) at T3, 16.67 (20.82) at T4, and 25.83 (26.16) at T5.

## Discussion

The present study explored the effects of MI on the functional rehabilitation of locomotor skills in persons with LLA. We tested the benefits of incorporating MI rapidly during the conventional physical therapy programs, hence assessing its therapeutic relevance during the acute stages post-amputation. The main results revealed an early positive impact of MI for the 10 m walking task during the pre-prosthetic phase, and slightly greater performance for the TUG during the prosthetic phase. The clinical measures of the amputated limb force further revealed greater performance at the end of the rehabilitation in the MI group, where the proportion of participants walking without any assistance was higher.

Participants who practiced MI outperformed those from the control group during the 10 m walking task during the pre-prosthetic phase. During this period, locomotor training with MI consisted in unilateral walking between the parallel bars with an emphasis on strong and safe ground contact with the intact limb. These exercises alternated with the balancing of the affected limb through hip flexion/extension, to simulate the movements as if participants were already walking with their prosthesis. This result therefore suggests that MI of unilateral walking during the acute phase following LLA is likely to promote prosthesis use as soon as participants can start wearing it. In the specific context of rehabilitation, Malouin et al. [9] examined results from several experimental studies on locomotor relearning in stroke persons and persons suffering from Parkinson disease. They suggested that MI at the initial phase of rehabilitation (when no physical training is possible) might foster functional rehabilitation by preparing participants for when they start to walk again. Even when performed alone, MI may thus improve motor learning through the activation of the neural networks involved in the actual execution of the skill [8]. In the specific case of amputation, the body schema, which corresponds to the internal representation of the location and orientation of body parts and their relative motion in space, is altered [44]. Such effect is emphasized in the absence of a prosthesis [45]. MI has been shown to promote acquisition of object’s use knowledge [46], which is sufficient to incorporate a tool to the body schema in healthy participants [47]. In a 71-year-old woman, who had suffered from transfemoral amputation seven years ago and still needed a standard walker for locomotor activities, Matalon et al. [28] also reported improved prosthesis embodiment (integration into the body schema) after a 4-week MI program including 3–8 min walking, balancing, and reaching tasks, three times per week. The participant reported that phantom sensations during MI helped her to improve limb location awareness and positioning, hence increasing trust in prosthesis strength and sense of motor control. Data further showed that, for the first time since her amputation, she was able to walk without external assistance. The present results support and extend these findings by highlighting the early positive impact of MI on walking ability during the acute phase of amputation. We thus postulate that performing MI of walking before prosthesis might foster easier prosthesis use directly after fitting. Although this beneficial effect of MI during the pre-prosthetic phase is promising, the difference between Control and MI groups may also result from differences in individual or functional abilities at the start. Such influence cannot here be totally ruled out due to the absence of baseline walking tests. Furthermore, while the participants included in this study exhibited comparable functionality scores at the start of the rehabilitation program, a slight age difference was observed in favor of the MI group. A potential influence of age differences on walking speed results can thus not be excluded.

The beneficial effects of pre-prosthetic MI observed on simple walking abilities did not, however, persist after prosthesis fitting. A simple explanation could be a ceiling effect, as participants from the MI group already displayed a level of performance after prosthesis fitting (21s) similar to that of the control group at the end of the rehabilitation program (22s). Another explanation might come from the means we used to assess walking performance, i.e. through the time required to perform the task. In their feasibility study, Vanmairis [48] observed that while MI did not impact walking speed per se, step length and limb-loading symmetries increased only in the MI group. Improvement of several walking parameters following MI were also reported in healthy participants who, with their knee bent, learnt to walk with an adapted version of a transfemoral prosthesis [49]. In this study, the authors compared the selective effectiveness of 1 session per week (for 3 weeks) of walking training through pure physical practice (30 min) or combined with MI (10 min of MI with 20 min of physical practice). Data revealed greater step length as well as walking security and quality improvements in the MI group. In the present study, MI of the 10 m walking task during the prosthetic phase may also have provided similar results in addition to the mere time of execution. Future studies should thus assess walking parameters such as step length and limb-loading symmetry to understand more deeply the positive effects of MI, and before drawing final conclusions.

The pattern of results was slightly different for the TUG, which did not reveal substantial pre-prosthetic MI benefits. Accordingly, data revealed that the MI and control groups exhibited similar levels of performance during the first evaluation after prosthesis fitting. However, contrarily to walking between the parallel bars, which started at the beginning of the pre-prosthetic phase of the rehabilitation program, participants rehearsed the TUG only once they were fitted with their prosthesis. Hence, the amount of MI training on this task was very limited and could explain the absence of initial results. Moreover, the effectiveness of MI has been shown to depend, at least partially, on MI quality. Neuroimaging results notably showed that good imagers recruit more efficiently brain regions involved in motor planning and execution [50], thus presuming fostering activity-dependent neuroplasticity and associated motor relearning outcomes. We assume that forming vivid and accurate MI of a complex functional task such as the TUG may have been too challenging. At this stage of the rehabilitation process, the absence of MI benefits on complex locomotor abilities recovery may also stem from participants’ low motor-control, as high level of motor expertise remains critical for optimum MI outcomes.

MI of the TUG did however result in higher performance gains from the time of prosthesis fitting to the time of rehabilitation program discharge. Specifically, the time required to perform the sequence of walking, turning, sitting, and raising movements, which required different aspects of fine balance and walking capacities, decreased more rapidly in participants who were subjected to MI training in addition to physical practice. These data are in agreement with previous findings supporting the benefits of MI on the TUG in a person with transtibial amputation [28]. In this single-case study, 12 MI sessions contributed to decrease the time to perform the TUG by 6s, which represented important clinical improvement [51]. Moreover, while the participant needed a standard walker to perform the task before MI, only a single point cane or stand-by assist (when no tool was used) remained necessary after MI training program. Vanmairis [48] also reported clinically important improvement on the TUG in four participants with transtibial amputations. The time required to perform the task decreased by 15.8s, almost twice the improvement of participants from the control group (8.5s), after 10 sessions of MI scheduled over two weeks.

Interestingly, our clinical measures of the amputated limb force further revealed greater performance in the MI group before discharge. The proportion of participants walking without any assistance was also higher at the end of the rehabilitation. These positive effects of MI were observed while the time of hospitalization was significantly reduced in the patients of the MI group, hence suggesting a more efficient and rapid recovery of motor functions. Taken together, present results tend to support the beneficial effects of prosthetic MI on both walking and motor performance in persons with LLA at a transtibial or transfemoral level.

After discharge, when no more MI training was provided, further performance gains were observed in participants from the control group, so that the final level of performance for the TUG was comparable in all participants. This result could be explained by a ceiling effect for participants in the MI group, who reached this level of performance more than twice faster (mean performance time = 13s) than the control group at the discharge time (mean performance time = 30s). Although the final level of performance was similar in the two groups, MI might thus contribute to accelerate the recovery process, and should then certainly be continued as a subsequent regular routine.

Although this study was specifically designed to investigate the impact of MI training on motor recovery, secondary beneficial effects on pain were somewhat expected. Spurred by results by MacIver et al. [24], who reported a significant reduction in pain intensity in persons with upper-limb amputation after a 6-week MI program based on the perception of the phantom limb, and the mental performance of comfortable and smooth movements, we expected a similar effect in our cohort. Data, however, did not support this assumption. This may be due to discrepancies between participants’ characteristics and both MI interventions. In the present study, participants were not recruited on the basis of elevated neuropathic pain levels (with a score systematically inferior to 30), and MI program exclusively focused on motor aspects. Moreover, pain remains a complex phenomenon involving central, peripheral, and psychological factors [52]. Specific studies looking at this issue should certainly consider in greater details the characterization of pain perception. For these reasons, present data do not allow drawing firm conclusions on potential effects of MI on pain following LLA.

As with the majority of studies, the design of the current study is subjected to limitations, specifically a small sample size and a lack of information on the level of functional mobility before amputation. Participants of the MI group were also slightly younger than those assigned to the control group, and included 4 persons amputated after a traumatic injury, who yielded greater motor recovery. Subdividing larger samples to analyze separately participants with regards to the origin of the trauma would be relevant. A possible difference at baseline measures is also a critical aspect that should have been more deeply considered as it may have contributed to explain some differences found when comparing the two groups. In particular, there was a slight age difference between the two groups, which may have influenced the self-selected walking speed for completing the locomotor tasks. Future studies should thus certainly control this variable of influence to replicate and confirm present findings. Although we did not cross the uncorrected statistical significance threshold when contrasting quantitative and qualitative baseline measures in both groups, we cannot firmly rule out potential baseline differences, particularly regarding the functional walking level. Noteworthy, the difference which approached the statistical significance threshold was in favor of the MI group, who might thus potentially have been a weaker responder to the MI intervention due to ceiling effects. Overall, a more thorough and complete procedure to establish comparability between groups at the moment of inclusion remains warranted to replicate the present findings, as is a randomized control trial, before drawing firm conclusions on the effectiveness of MI in persons with LLA. Finally, and although we used manipulation checks to control adherence to imagery guidelines and to collect self-report-ratings of imagery quality, additional information, such as standardized verbal description from participants, might be gathered to control MI practice.

## Conclusion

Current findings highlighted some beneficial effects of pre- and prosthetic phase MI on locomotor-related capacities and motor performance in a cohort of 19 persons with transtibial or transfemoral amputations. While a thorough assessment of participants’ physical condition and residual of motor functions before beginning the intervention remains necessary before concluding on the positive impact of MI, our findings tend to support the promising integration of MI in the classical course of physical therapy. Accordingly, we suggest that MI should be considered as an adjunct to current clinical interventions, as soon as possible in the motor recovery process, both to promote prosthesis use and amplify its recovering effect on functional movements. Performing detailed biomechanical analyses of walking in future studies should contribute to a better understanding of MI impacts during the pre- and prosthetic phases following LLA. From a more fundamental perspective, and while future experimental research might certainly provide a larger monitoring of neurophysiological data, the present findings support the critical importance of exploring motor imagery as a neurocognitive stimulation for patients.

### Electronic supplementary material

Below is the link to the electronic supplementary material.

Supplementary Material 1

Supplementary Material 2

## Data Availability

The data associated with the paper are not publicly available but remain available from the corresponding author on reasonable request.

## Abbreviations

ART: Aligned rank transformation
KVIQ: Kinesthetic and visual imagery questionnaire
MI: Motor imagery
TUG: Time up and go
LLA: Lower limb amputation
