# Supplementary material for: Towards efficient motor imagery interventions after lower-limb amputation
Source: J Neuroeng Rehabil. 2024 Apr 15;21:55. doi: 10.1186/s12984-024-01348-3 (PMC11017566; doi:10.1186/s12984-024-01348-3)
Supplement: Supplementary file 1 — Supplementary Material 1 [file 12984_2024_1348_MOESM1_ESM.docx]

Highlights

- Motor imagery improved 10m walking, TUG performance, and amputated limb force.
- A greater proportion of persons walked without assistance after imagery training.
- The amputated limb force revealed greater performance in the motor imagery group.
- Findings support integration of motor imagery in the course of physical therapy.
